# Prognostic utility of red blood cell distribution width-based ratios and their individual components for mortality prediction in dogs with acute pancreatitis

**DOI:** 10.3389/fvets.2026.1927456

**Published:** 2026-09-22

**Authors:** Alba Diaz-Gonzalez, Lorena Espadas-Gonzalez, Eva M. Pérez-Merino, Carlos Martínez-Zafra, Jose Ignacio Cristóbal-Verdejo

**Affiliations:** Departamento de Medicina Animal, Hospital Clínico Veterinario, Facultad de Veterinaria, Universidad de Extremadura, Cáceres, Spain

**Keywords:** canine acute pancreatitis, hematological ratios, hemoglobin, prognostic biomarkers, red blood cell distribution width

## Abstract

**Introduction:**

Prognostic assessment is critical in dogs with acute pancreatitis (AP). Composite biomarkers such as the red blood cell distribution width (RDW)-to-albumin ratio (RAR) and hemoglobin-to-RDW ratio (HRR) have shown prognostic value in human critical care but have not been evaluated in canine AP.

**Methods:**

A retrospective cohort study was conducted in 80 dogs with AP and 10 healthy controls. The diagnostic and prognostic utility of admission HRR and RAR was compared with that of their individual components measured at admission (RDW, hemoglobin and albumin). Outcome was classified as survival or non-survival to hospital discharge. Receiver operating characteristic (ROC) curve analysis and multivariable logistic regression were performed.

**Results:**

The overall mortality rate was 33.75%. Non-survivors presented with significantly lower hemoglobin, albumin and HRR values and significantly higher RDW values than survivors, whereas RAR did not differ between outcome groups. Among the variables evaluated, hemoglobin showed the highest discriminatory performance (AUC = 0.704; 95% CI: 0.583–0.812), followed by HRR (AUC = 0.669; 95% CI: 0.547–0.790), RDW (AUC = 0.663; 95% CI: 0.540–0.786), CAPCSI (AUC = 0.658) and albumin (AUC = 0.634; 95% CI: 0.506–0.757). In the multivariable model including age, hemoglobin, albumin, RDW and CAPCSI, only CAPCSI remained independently associated with survival (adjusted OR = 0.612; 95% CI: 0.403–0.929; *p* = 0.021).

**Discussion:**

Integrating erythrocyte indices into composite ratios offered no clinical advantage over evaluating individual markers in dogs with acute pancreatitis. Among the laboratory variables assessed, hemoglobin showed the highest discriminatory performance, although its overall prognostic accuracy remained moderate. These findings suggest that erythrocyte-derived variables may serve as adjunctive prognostic markers in dogs with AP but should be interpreted alongside established clinical assessment.

## 1 Introduction

Canine acute pancreatitis (AP) is an inflammatory condition characterized by neutrophilic inflammation, necrosis, and edema. Its severity ranges from mild to life-threatening; therefore, early detection and accurate prognostic evaluation are critical for guiding timely therapeutic strategies and clinical monitoring (1).

While the diagnosis of canine AP relies on a combination of clinical, laboratory, and imaging findings, the assessment of disease severity remains challenging, particularly in the early stages (2). Consequently, research has increasingly focused on routine biomarkers. Red blood cell distribution width (RDW), a parameter quantifying anisocytosis (3), is widely used to assess inflammatory (4), cardiovascular (5), neoplastic conditions (6, 7), and acute pancreatitis (8–10) in human medicine.

Although RDW has shown diagnostic and prognostic potential in veterinary medicine (11), its use for evaluating canine AP remains controversial (12, 13).

Systemic inflammation in AP frequently triggers severe complications, including hypoalbuminemia and anemia. Hypoalbuminemia may result from a negative acute-phase response and increased vascular permeability (14), whereas anemia has been associated with oxidative stress and potential hemolysis (15, 16). Although hypoalbuminemia is a well-established prognostic factor in critically ill dogs (17–19), the prognostic value of hemoglobin levels in canine AP is less well characterized than that in humans (15).

In human studies, composite indices, such as the RDW-to-albumin ratio (RAR), have shown prognostic promise for thromboembolic diseases (20, 21) and sepsis, whereas the hemoglobin-to-RDW ratio (HRR) has demonstrated utility in other critical conditions (22). However, these composite ratios remain unexplored in the veterinary literature.

The objective of this study was to evaluate the diagnostic and prognostic utility of the HRR and RAR in dogs with AP and compare their performance with their individual components (RDW, albumin, and hemoglobin) in order to identify the most reliable mortality marker in this population.

## 2 Materials and methods

### 2.1 Animals

The medical records of dogs diagnosed with AP at the Veterinary Teaching Hospital of the University of Extremadura from 2021 to 2025 were reviewed for this retrospective cohort study. The Hospital board approved this study, and written informed consent was obtained from all owners.

Elevated canine pancreatic lipase activity levels above the laboratory upper reference limit (>160 U/L), measured via the v-LIP-P assay, combined with supportive clinical and ultrasonographic findings, served as the basis for the diagnosis of AP. The inclusion criteria were as follows: complete hematological and serum biochemical data collected within 24 h of presentation and before treatment initiation, along with follow-up information spanning at least 1 month to rule out concurrent illnesses. Although higher cutoffs (e.g., 2–three times the upper limit) are often recommended when evaluating lipase activity in isolation (23), we mitigated the risk of non-specific increases by strictly requiring an acute onset of at least two clinical signs (vomiting, diarrhea, anorexia, abdominal pain, or lethargy) and ultrasound evidence of pancreatic involvement (enlargement, hypoechoic parenchyma, hyperechoic peripancreatic fat, or peripancreatic effusion) (2, 24, 25). The exclusion criteria included incomplete medical records, concurrent illnesses, excess glucocorticoids (endogenous or exogenous), history of recurrent AP episodes, and clinical signs present for more than 7 days.

Since HRR and RAR have not previously been reported in dogs, 10 dogs (five males and five females) scheduled for routine sterilization were recruited to provide descriptive comparative data. Health status was confirmed through clinical history, physical examination and haematobiochemical profiles. This group was not intended to establish reference intervals, which would require a substantially larger population.

### 2.2 Data

The data collected included signalment variables (sex, age, and breed), length of hospitalization, and disease severity, which were assessed using the canine acute pancreatitis clinical severity index (CAPCSI), originally developed by Mansfield et al. (26) and scoring cardiac, respiratory, intestinal integrity and blood pressure parameters (19). The score ranges from 0 to 10 points, with higher values indicating more severe disease. All components required for its calculation were available in the medical records of the dogs included, as complete clinical and laboratory data at admission were among the inclusion criteria.

On admission, all dogs underwent a thorough clinical examination. Blood samples were collected at presentation, before the administration of fluid therapy or any other treatment. Complete blood counts were performed using a ProCyte DX^®^ hematology analyzer (IDEXX Laboratories, USA). Serum biochemical analyses were conducted using a Spin200E automated analyzer (Spinreact^®^, Barcelona, Spain). Pancreatic lipase activity was measured using the v-LIP-P slide on a FUJI DRI-CHEM 4000i analyzer (Fujifilm^®^, Tokyo, Japan).

The recorded laboratory variables included serum albumin concentration (g/dl), blood hemoglobin concentration (g/dl), and RDW (%).


$$\begin{array}{lll} RAR & = & \frac{RDW}{serum\;albumin}\\ HRR & = & \frac{blood\;hemoglobin}{RDW} \end{array}$$

The dogs were classified according to their survival status at the end of the hospitalization period. Those discharged alive were considered survivors, whereas those who died or were euthanised during hospitalization were classified as non-survivors. In accordance with the ethical standards of our institution, euthanasia was never chosen for financial reasons. It was performed strictly based on clinical criteria to prevent further refractory suffering despite intensive medical therapy.

### 2.3 Statistical analysis

Statistical analyses were performed using IBM SPSS Statistics (version 29.0; IBM Corp., Armonk, NY, USA). The normality of the continuous variables was evaluated using the Kolmogorov–Smirnov or Shapiro–Wilk tests, as appropriate. Normally distributed variables are presented as the mean ± standard deviation, whereas non-normally distributed variables are expressed as the median and range (minimum-maximum).

Group comparisons were conducted using Student's *t*-test or the Mann–Whitney U test for normally or non-normally distributed continuous variables, respectively. Categorical variables (e.g., sex) were compared using the chi-square or Fisher's exact tests, as appropriate. The correlation among the ratios, laboratory analytes, and clinical scores was assessed using Spearman's rank or Pearson's correlations, depending on the data distribution.

To evaluate the trend in mortality risk across different biomarker levels, variables were categorized into quartiles. The association between quartiles and mortality prevalence was analyzed using the chi-square test and Somers'D statistic to assess the directional association.

The discriminatory power of the analytes, ratios and CAPCSI was assessed using the area under the receiver operating characteristic curve (AUROC). The optimal cut-off values were determined using the Youden index. Sensitivity, specificity, odds ratios (ORs), and their respective 95% confidence intervals (CIs) were calculated to determine the diagnostic accuracy and predicted risk of death.

Multivariable binary logistic regression was performed to assess the independent associations of hemoglobin, albumin, RDW, CAPCSI, and age with survival.

Statistical significance was set at *p* < 0.05.

## 3 Results

Based on the exclusion criteria, 34 dogs were excluded from this study (16 due to concurrent illness, six due to prior corticosteroid therapy, and 12 due to lack of data), resulting in a final sample of 80 dogs. Of these, breed information was available for 77 dogs; 58 were purebred dogs, mostly Yorkshire Terriers (*n* = 11) and Ratonero Bodeguero Andaluz (*n* = 7), and 19 were mixed-breed dogs. The study population included 44 males and 36 females, with a median age of 10 years.

The control group (*n* = 10) comprised five mixed-breed and five purebred dogs, with five males and five females, and a mean age of 3.5 years. Neither group showed significant differences in breed (*p* = 0.420) or sex distribution (*p* = 0.742). However, healthy dogs were significantly younger than those with AP (*p* < 0.001). As shown in Table 1, the AP group exhibited significantly lower concentrations of hemoglobin, albumin, and HRR than the control group. Conversely, the RAR was significantly higher in dogs with pancreatitis. RDW was the only parameter that did not differ significantly between groups.

**Table 1:** Admission hematological markers of the control group of healthy dogs and dogs with acute pancreatitis (AP).

Parameter	Control group (*n* = 10)	AP dogs (*n* = 80)	*p*-value
Age (years)	3.5 (1.5–9.5)	10.0 (1–18)	< 0.001
RDW (%) (RI: 13.6–21.7)	16.4 (15.6–18)	17.18 (13.2–27.1)	0.775
Hemoglobin (g/dl) (RI:13.1–20.5)	17.25 (14.97–17.97)	12.8 (9.5–15.3)	0.01
Albumin (g/dl) (RI: 2.5–3.9)	3.2 (3.08–3.23)	2.6 (2.4–3)	< 0.001
HRR	1.05 (0.68–1.29)	0.76 (0.21–1.37)	0.002
RAR	5.1 (4.36–5.93)	6.37 (4.03–12.76)	0.001

RI, reference interval; RDW, red blood cell distribution width, HRR, hemoglobin-to-RDW ratio, RAR, RDW-to-albumin ratio. Data are shown as the median and range.

The median interval from the onset of clinical signs to admission was 4 days (0–7). On admission, the median serum pancreatic lipase activity was 1,590 U/L (310–7,200).

Abdominal pain was recorded in 75 dogs (93.8%), and anorexia was noted in 73 (91.3%). Vomiting was observed in 71 dogs (88.8%), whereas diarrhea was observed in 59 (73.8%). No dogs presented with haematemesis. Haemorrhagic diarrhea was recorded in four dogs (5.0%). No significant associations were found between the presence of macroscopic overt haemorrhagic signs and the admission values of hemoglobin (*p* = 0.44), albumin (*p* = 0.732), or RDW (*p* = 0.895). Lethargy was detected in 58 dogs (72.5%), and signs suggestive of nausea were identified in 47 (58.8%). Since the acute onset of at least two of these signs was required for inclusion, the following frequencies describe the study population rather than the prevalence of these signs in canine AP.

Pancreatic enlargement was identified in 36 dogs (45.0%). The pancreatic parenchyma was hypoechoic in 72 dogs (90.0%) and hyperechoic in six (7.5%); parenchymal heterogeneity was noted in 59 dogs (73.8%). A hyperechoic mesentery was observed in 73 dogs (91.3%), whereas no dogs presented with hypoechoic mesentery. Peripancreatic free fluid was identified in 13 dogs (16.3%), and pancreatic cysts or pseudocysts occurred in two (2.5%).

The median CAPCSI score for the study population was four (1–7). The distribution of CAPCSI scores among dogs with AP was as follows: CAPCSI 1, *n* = 4 (5%); CAPCSI 2, *n* = 9 (11.25%); CAPCSI 3, *n* = 26 (32.5%); CAPCSI 4, *n* = 15 (18.75%); CAPCSI 5, *n* = 18 (22.5%); CAPCSI 6, *n* = 6 (7.5%); and CAPCSI 7, *n* = 2 (2.5%). No dogs had CAPCSI scores >7.

All patients received intravenous fluid therapy at individually adjusted rates based on hydration deficits and fluid loss. Regarding supportive care, antiemetic treatment with maropitant was administered to 80 dogs (100%) and combined with ondansetron in 51 (63.8%). When further control of vomiting was necessary, a continuous infusion of metoclopramide was administered to 17 dogs (21.3%). Gastroprotectants were used in 15 dogs (18.8%), and probiotics were prescribed for diarrhea management in 59 (73.8%). Analgesia was initially established with opioids (buprenorphine or methadone) in 75 dogs (93.8%). To achieve adequate pain control, metamizole was required in 61 dogs (76.3%), and was used as the sole analgesic in five (6.3%). Antibiotic therapy (amoxicillin–clavulanic acid) was administered to 21 dogs (26.3%) exhibiting signs of sepsis. Nutritional support relied on a high-quality, low-fat wet diet. In some instances, the owners declined the recommendation for feeding tube placement.

Regarding clinical outcomes, 53 dogs (66.25%) survived until discharge, whereas 27 (33.75%) died (*n* = 22; 27.5%) or were euthanised (*n* = 5; 6.25%) during hospitalization. Comparisons between survivors and non-survivors revealed no statistically significant differences in age (*p* = 0.455), sex distribution (*p* = 0.344), or the duration of clinical signs before admission [3 (0–6) vs. 4 days (0–7); *p* = 0.179]. Similarly, the serum pancreatic lipase activity did not differ significantly between groups (*p* = 0.075), although the median values were higher in non-survivors [1,835 U/L (680–6,600)] compared with those in survivors [954 U/L (310–7,200)]. In contrast, the CAPCSI score was a significant differentiating factor (*p* = 0.015); non-survivors presented with significantly higher severity scores [median 4 (2–7)] than survivors [median 3 (1–7)] (Figure 1). No significant differences were observed (*p* = 0.38) in the duration of hospitalization between survivors [median 5 days (1–12)] and non-survivors [median 4 days (1–10)].

**Figure 1:**
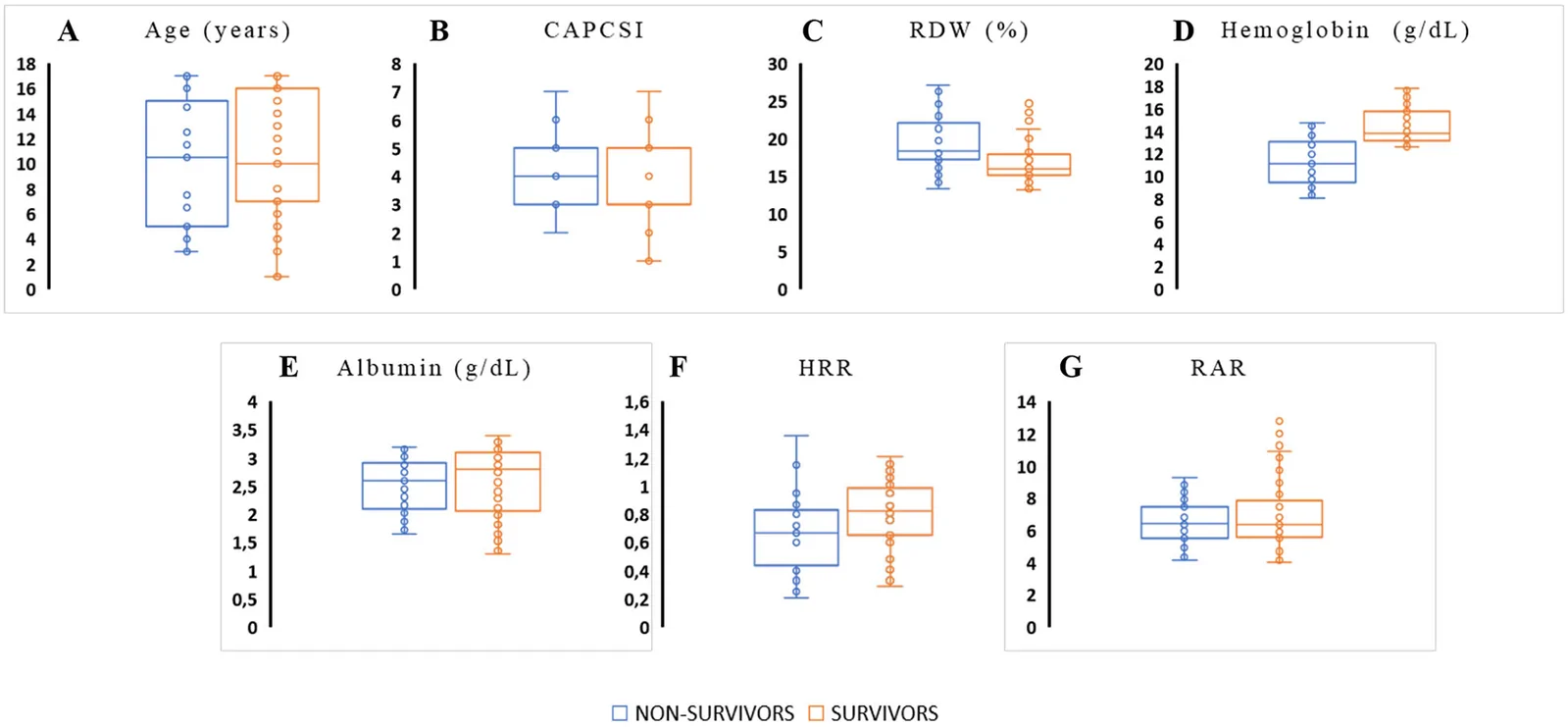
Distribution of clinical and hematological variables according to survival outcome in dogs with acute pancreatitis. Box-and-whisker plots showing age **(A)**, Canine Acute Pancreatitis Clinical Severity Index (CAPCSI) **(B)**, red blood cell distribution width (RDW) **(C)**, hemoglobin concentration **(D)**, albumin concentration **(E)**, hemoglobin-to-RDW ratio (HRR) **(F)**, and RDW-to-albumin ratio (RAR) **(G)** in survivors and non-survivors.

Table 2 compares admission laboratory markers between the survivors and non-survivors. Surviving dogs exhibited significantly higher median hemoglobin and serum albumin levels than non-survivors, whereas non-survivors exhibited significantly higher RDW values. Regarding the composite indices, the calculated HRR was significantly lower in the non-survivor group. No statistically significant difference was found in the RAR values between the two groups.

**Table 2:** Admission hematological marker results for surviving and non-surviving dogs with acute pancreatitis.

Parameter	Survivors (*n* = 53)	Non-survivors (*n* = 27)	*p*-value
Age (years)	10 (1–17)	10.5 (3–17)	0.455
RDW (%)	16.05 (13.2–24.7)	18.3 (13.3–27.1)	0.015
Hemoglobin (g/dl)	13.8 (12.6–17.8)	11.1 (8.08–14.75)	0.001
Albumin (g/dl)	2.8 (1.3–3.4)	2.6 (1.65–3.2)	0.045
HRR	0.82 (0.29–1.21)	0.67 (0.21–1.36)	0.012
RAR	6.33 (4.03–12.77)	6.42 (4.18–9.29)	0.797

RDW, red blood cell distribution width; HRR, hemoglobin-to-RDW ratio; RAR, RDW-to-albumin ratio.

Data are shown as median and range.

Analysis of mortality prevalence across biomarker quartiles (Table 3) revealed a significant ordinal association between increasing RDW categories and mortality (Somers' D, *p* = 0.001), together with a significant linear trend across quartiles (χ^2^, *p* = 0.007). For HRR, mortality was highest in the lowest quartile and progressively decreased across higher quartiles. Although differences in mortality prevalence between quartiles did not reach statistical significance (χ^2^, *p* = 0.06), a significant ordinal association between increasing HRR values and lower mortality was observed (Somers' D, *p* = 0.005). In contrast, RAR showed neither significant differences in mortality prevalence across quartiles (χ^2^, *p* = 0.34) nor a significant ordinal association with mortality (Somers' D, *p* = 0.72).

**Table 3:** Prevalence of mortality in dogs with acute pancreatitis by hematological marker quartiles.

Parameter	Measure	Quartile 1	Quartile 2	Quartile 3	Quartile 4	*p*-value
RDW (%)	Value	< 15.15	17.18	18.9	>20.54	χ^2^ = 0.007; *D* = 0.001
	% mortality	13.3	22.2	39.1	66.7	
	*n* (death/total)	2/15	6/27	9/23	10/15	
HRR	Value	< 0.6	0.75	0.94	>1.08	χ^2^ = 0.06; *D* = 0.005
	% mortality	54.5	35	27.3	18.5	
	*n* (death/total)	12/22	7/20	3/11	5/27	
RAR	Value	5.52	6.38	7.47	>8.57	χ^2^ = 0.34*; D* = 0.72
	% mortality	38.5	28.6	47.6	22.2	
	*n* (death/total)	5/13	8/28	10/21	4/18	

RDW, red blood cell distribution width; HRR, hemoglobin-to-RDW ratio; RAR, RDW-to-albumin ratio.

*p*-values correspond to Chi-squared test (χ^2^) and Somers' D (D) test.

Table 4 and Figure 2 present the results of the AUROC curve analysis with optimal cutoff values for the statistically significant parameters used to distinguish non-survivors and survivors. When evaluating individual laboratory analytes, hemoglobin levels exhibited the highest discriminatory ability (AUC = 0.704), followed by RDW (AUC = 0.663) and albumin (AUC = 0.634). Regarding the derived ratios, HRR exhibited moderate discriminative ability, with an AUC of 0.669 (*p* = 0.012). Values above the cut-off for RDW and below the cut-off for hemoglobin, albumin, and HRR were significantly associated with increased odds of death. RDW demonstrated the highest specificity (86.4%) among the individual markers tested (Table 4).

**Table 4:** Receiver operating characteristic curve analysis, showing the overall accuracies of the hematological-derived markers used to distinguish survival from non-survival dogs with acute pancreatitis.

Parameter	AUC (95% CI)	*P*	Cutoff	Sensitivity (%)	Specificity (%)	Odd ratio (95% CI)	*p*-value
RDW (%)	0.663 (0.540–0.786)	0.015	18.85	42.4 (27.3–59.2)	86.4 (72.8–93.9)	4.66 (1.54–14.07)	0.004
Hemoglobin	0.704 (0.583–0.812)	0.001	11.3	52.3 (36.4–68.2)	84.8 (72.7–97)	6.13 (2–18.81)	< 0.001
Albumin	0.634 (0.506–0.757)	0.045	2.6	59.1 (45.5–72.7)	66.7 (51.5–81.8)	2.89 (1.13–7.4)	0.038
HRR	0.669 (0.547–0.790)	0.012	0.66	50 (35.9–64.1)	84.8 (68.5–93.7)	5.6 (1.82–17.16)	0.002

AUC, area under the receiver operating characteristic curve; RDW, red blood cell distribution width; HRR, hemoglobin-to-RDW ratio; RAR, RDW-to-albumin ratio.

*p*-values correspond to the chi-squared test. Mortality was associated with values above the cut-off for RDW and below the cut-off for hemoglobin, albumin, and HRR.

**Figure 2:**
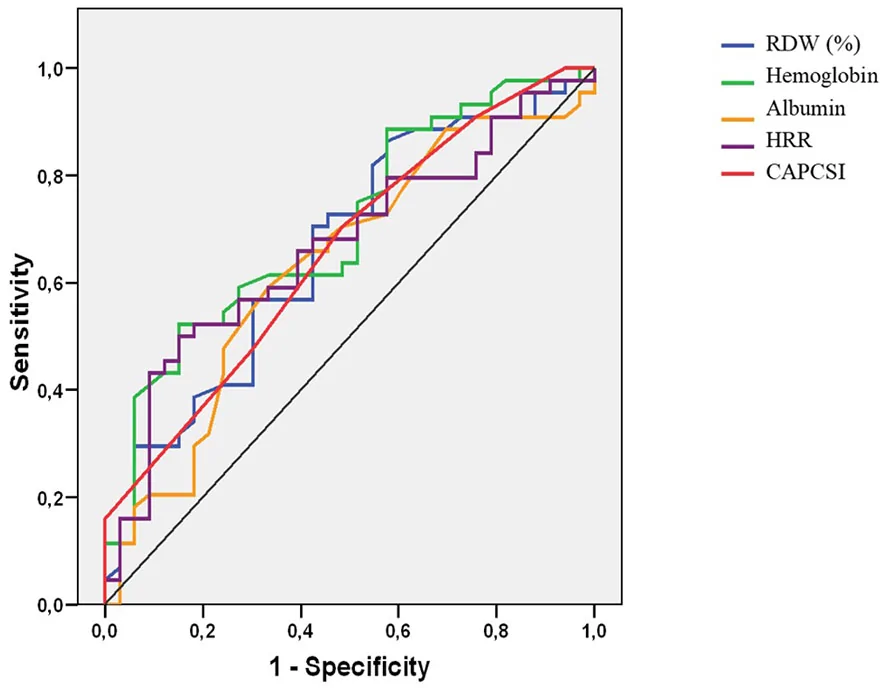
Receiver operating characteristic (ROC) curves comparing the prognostic performance of Canine Acute Pancreatitis Clinical Severity Index (CAPCSI), red blood cell distribution width (RDW), hemoglobin concentration, albumin concentration and hemoglobin-to-RDW ratio (HRR) for predicting in-hospital mortality in dogs with acute pancreatitis. Non-survival was associated with values above the cut-off for RDW and below the cut-off for hemoglobin, albumin, and HRR.

ROC curve analysis yielded an AUC of 0.658 (95% CI: 0.536–0.780; *p* = 0.018) for CAPCSI and identified an optimal cutoff value of 4. At this threshold, sensitivity and specificity for predicting non-survival were 70.5% (55.6–81.9) and 51.5% (35.2–67.5), respectively. CAPCSI scores above this threshold yielded an odds ratio for death of 2.53 (95% CI: 0.99–6.49); however, the association was not significant (*p* = 0.053).

The multivariable logistic regression model was significant (*p* = 0.002). After adjustment for hemoglobin, albumin, RDW, and age, CAPCSI was the only variable independently associated with survival (adjusted OR = 0.612, 95% CI 0.403–0.929; *p* = 0.021), whereas the remaining variables were not significant (all *p* > 0.05).

Additionally, no significant correlations were observed between the CAPCSI scores and hematological parameters analyzed (*p* = 0.12).

## 4 Discussion

We hypothesized that combining the RDW value with hemoglobin and albumin levels would improve the prognostic accuracy for canine AP. Although admission hemoglobin exhibited the highest discriminatory performance among the laboratory variables evaluated, its overall prognostic accuracy remained moderate. Similarly, HRR was associated with outcome but did not provide a clear advantage over its individual components, whereas RAR showed no prognostic utility in this cohort. Although RDW has demonstrated conflicting prognostic utility in veterinary literature (12, 13), this is the first study to evaluate these erythrocyte-based ratios in dogs.

The 33.75% mortality rate in our cohort aligns with previously published ranges of 33–40.6% (13, 17, 18, 27). The contrast with the lower mortality rate reported recently (8.8%) (28) may reflect differences in disease severity at presentation, as indicated by the higher CAPCSI scores observed in our cohort. Additionally, the longer duration of clinical signs prior to admission compared with that reported in other studies (23) may have contributed to the increased mortality observed. Non-survivors included dogs that died and dogs euthanised on clinical and welfare grounds, the latter only in patients whose disease had reached a terminal course. This highlights the need for routine biomarkers to guide early management and inform owners about prognosis.

We selected biomarkers derived from routine analytes, that is, erythrocyte indices and serum albumin levels, as they offer daily clinical applicability. RDW has established prognostic value in human acute pancreatitis (8, 29) and several canine conditions (11, 30, 31). The association between elevated RDW and mortality is driven by systemic inflammation, which disrupts iron metabolism and bone marrow function. Oxidative stress alters the integrity of red blood cell membranes and immature cells may reach the circulation, both of which increase morphological heterogeneity. Pro-inflammatory cytokines simultaneously impair iron availability and suppress erythroid precursors, reducing the output of new cells. Together, these processes increase morphological heterogeneity and RDW during severe inflammation (3, 32).

In this study, RDW did not differ significantly between the AP group and healthy controls, with the median values for both cohorts remaining within the interval reported by the analyser manufacturer. This lack of diagnostic differentiation has also been observed in other conditions, such as canine myxomatous mitral valve disease (33). Inflammation rather suppresses erythroid production, as occurs in anemia of inflammatory disease, and may therefore also lead to decreased RDW (32). This could explain the lack of difference between control and AP dogs observed and is consistent with the reduced hemoglobin concentrations recorded in our cohort. Because previous canine AP studies lacked control groups (12, 13), our findings suggest that RDW can serve as a prognostic indicator of severity, rather than as a diagnostic biomarker.

The median RDW in our overall AP cohort [17.18 (13.2–27.1)], as well as in survivors [16.05 (13.2–24.7)] and non-survivors [18.3 (13.3–27.1)], aligned closely with those reported by Johnson et al. (13). However, Guglielmini et al. (12) reported notably lower medians across all groups (overall, 13.1; survivors, 12.5; non-survivors, 14.2). Despite this similarity to the findings of Johnson et al. (13), our statistical results paralleled those of Guglielmini et al. (12); we observed a significant difference in RDW between survivors and non-survivors, corroborating their results, whereas Johnson et al. reported no association.

These absolute RDW variations likely originated from instrumentation: Guglielmini et al. (12) used the Advia system (associated with lower ranges), whereas both our study and Johnson et al. (13) utilized the ProCyte Dx. Although Johnson et al. (13) defined >14.5% as elevated, we observed mortality risk at values still within the manufacturer's limits (13.6–21.7%). This finding highlights that prognostic thresholds may differ from the analytical thresholds used to define abnormality. Consequently, RDW values considered normal at admission may nevertheless carry prognostic information and be associated with differences in clinical outcome.

Although Johnson et al. (13) did not identify a predictive cutoff for RDW, our findings align with the prognostic utility proposed by Guglielmini et al. (12), who reported a five-fold increase in mortality risk for RDW >12.7% (OR 5.08; 95% CI 1.14–22.67). In our cohort, a cutoff of >18.85 yielded comparable risk (OR 4.66; 95% CI 1.54–14.07). This association was further supported by the quartile analysis, which demonstrated a significant increase in mortality across progressively higher RDW categories together with a significant ordinal association between increasing RDW and mortality.

Low hemoglobin levels have been found to predict severity and mortality in human AP (34), critically ill dogs (35), and other canine systemic conditions (36, 37). Despite its clinical relevance, data on canine AP are limited to a single recent study that associated lower hemoglobin concentrations with poorer outcomes (15). Our findings support this association, although the overall hemoglobin concentrations observed in our cohort differed from those reported by Chawanlawuthi et al. (15), likely reflecting differences in case severity at presentation.

The decline in hemoglobin in dogs with AP is likely multifactorial and may encompass gastrointestinal hemorrhage (1), inflammatory disruption of iron metabolism and erythropoiesis (32), and secondary immune-mediated hemolysis (16).

Building on the individual prognostic values of RDW and hemoglobin, we evaluated their integration into the HRR. Although the HRR is a recognized prognostic factor for various human systemic diseases (22, 38–40), to the best of our knowledge, this is the first study to explore its clinical utility in veterinary medicine. The only human AP study evaluating HRR (41) reported significantly lower admission values in non-survivors than in survivors, as observed in our canine cohort. The association with non-survival was further supported by the quartile analysis, which demonstrated a significant ordinal association between decreasing HRR values and increasing mortality. Reduced HRR values reflect the combined effects of anemia and anisocytosis. Given that overt haemorrhagic signs were infrequent and were not associated with admission hemoglobin, albumin, or RDW values in our cohort, the observed reduction in hemoglobin and HRR may be more consistent with inflammatory mechanisms (12, 32) than with overt gastrointestinal blood loss (42), although the small number of dogs with haemorrhagic signs precludes definitive conclusions. Lower hemoglobin concentrations may reflect the combined effects of inflammation-associated alterations in erythropoiesis and reduced oxygen-carrying capacity during the systemic inflammatory response associated with AP, thereby contributing to tissue hypoxia (43) and poorer outcome (34).

The albumin findings in our cohort paralleled those reported in people (44, 45) and in dogs (14) with AP, in which hypoalbuminaemia reflects a negative acute-phase response and the hypercatabolism driven by systemic inflammation (45–47). Consistent with previous studies (18, 19), non-survivors exhibited lower serum concentrations. Specifically, dogs falling below the 2.6 g/dl mortality threshold identified by Fabrès et al. (17) exhibited a nearly three-fold increased risk of death.

In human medicine, composite ratios combining inflammatory and nutritional markers have been extensively investigated. RAR is an established predictor of mortality and disease severity in several human critical care settings, including intensive care unit patients (48), sepsis (49), pneumonia (50), and myocardial infarction (51). In human AP, elevated RAR at admission predicts short-term mortality (48, 52). Despite its validity in human critical care, the clinical utility of RAR in veterinary medicine remains unexplored. In our cohort, RAR differed between healthy controls and dogs with AP, suggesting that the ratio reflects the inflammatory changes associated with the disease. However, unlike the findings reported in humans, RAR did not differ significantly between survivors and non-survivors, and neither significant differences across quartiles nor a significant ordinal association with mortality were observed.

Taken together, these findings indicate that RDW-based composite ratios did not improve prognostic performance relative to the individual variables from which they were derived. RAR offered no prognostic value despite both RDW and albumin being individually associated with mortality. The lack of prognostic utility of RAR may be influenced by factors such as hydration status and altered albumin concentrations (53). HRR, although associated with mortality, did not demonstrate greater discriminatory ability than hemoglobin alone. This is unsurprising given that the variation in RDW between survivors and non-survivors was modest relative to that observed for hemoglobin, suggesting that the prognostic behavior of HRR is largely determined by its hemoglobin component. Consequently, the additional calculation of these derived ratios appears to provide limited clinical benefit beyond the direct interpretation of routinely available hematological variables.

Biologically, reduced hemoglobin concentrations may compromise oxygen delivery and exacerbate tissue hypoxia during the hypermetabolic state associated with AP, providing a plausible explanation for its stronger prognostic performance.

According to the AUROC analysis, both RDW and HRR demonstrated only moderate discriminatory ability in the present study. Few studies have used ROC curve analysis to evaluate the discriminatory ability of these biomarkers in AP. In human medicine, HRR has shown a similarly moderate ability to predict cardiovascular mortality (54). In veterinary medicine, Guglielmini et al. (12) reported an AUC of 0.74 (95% CI 0.63–0.84) for RDW in canine AP, which was slightly higher than the value observed in our cohort (0.663; 95% CI, 0.540–0.786); however, the estimates and their confidence intervals overlapped.

In the present study, these erythrocyte-based markers demonstrated high specificity but moderate sensitivity (42.4–52.3%). Specificity was consistently high across the predictors, with RDW exhibiting the highest level (86.4%), followed by hemoglobin and HRR (84.8%). Consequently, although RDW values above the cut-off and hemoglobin, albumin and HRR values below their respective cut-offs were associated with non-survival, the moderate sensitivities indicate that many dogs that ultimately died would not have been identified at admission. Therefore, values falling on the lower-risk side of these thresholds should not be interpreted as evidence of a favorable prognosis.

In addition to erythrocyte-derived variables, several inflammatory biomarkers have been evaluated for prognostic purposes in canine AP. Neutrophil-based indices, including neutrophil-to-lymphocyte ratio and, more recently, neutrophil-to-albumin ratio, neutrophil percentage-to-albumin ratio, and neutrophil-to-lymphocyte ratio-to-albumin ratio, have shown moderate prognostic performance, with reported AUROC values ranging from 0.609 to 0.708 (13, 18).

In our previous study, NAR achieved the highest AUROC among these inflammatory indices (0.708) (18), a value very similar to the AUROC obtained for hemoglobin in the present cohort. Likewise, acute-phase proteins such as CRP have been associated with disease progression and outcome in canine AP. Keany et al. reported a positive correlation between CRP concentrations and clinical disease activity during hospitalization (19), whereas Oberholtzer et al. (55) found that dogs with CRP concentrations above 10 mg/L had a greater than five-fold increased risk of death. Interestingly, these biomarker classes appear to reflect different pathophysiological aspects of the disease. Neutrophil-based indices primarily reflect leukocyte activation and the magnitude of the inflammatory response (56), whereas acute-phase proteins such as CRP reflect hepatic acute-phase stimulation. In contrast, hemoglobin, RDW and HRR may reflect downstream consequences of systemic inflammation, including altered iron metabolism, impaired erythropoiesis, oxidative injury and reduced physiological reserve. Despite these biological differences, the prognostic performance reported for these biomarker groups is broadly similar. This suggests that no currently available laboratory marker fully captures the complexity of disease progression in canine AP and that different biomarkers may provide complementary information regarding the host response to pancreatic inflammation.

Hospitalization duration did not differ significantly between survivors and non-survivors, nor did it correlate with hematological ratios. Thus, RDW-based indices specifically reflected mortality risk, rather than the clinical recovery time.

CAPCSI was selected among other clinical severity scoring indices for canine AP because its components were consistently available in our retrospective records. CAPCSI scores were higher in non-survivors than in survivors. This finding differs from that reported by Keany et al. (19) and Cridge et al. (28), who did not identify significant differences in CSI scores between survivors and non-survivors. In the present study, ROC curve analysis revealed a moderate discriminatory performance (AUC = 0.658), similar to that observed for hemoglobin, RDW, and HRR. However, the association between CAPCSI scores above the identified cut-off and increased odds of death did not reach statistical significance. This suggests that, while CAPCSI retained an overall ability to discriminate between survivors and non-survivors, the prognostic value of the proposed threshold should be interpreted with caution. Cridge et al. (28) also reported only limited discriminatory ability for the score (AUC 0.537; 95% CI 0.446–0.629). Furthermore, in our study, CAPCSI was the only variable that remained independently associated with survival in the multivariable analysis performed on this cohort. The inconsistent findings across studies may reflect differences in the characteristics of the study populations, disease severity, and mortality rates, as our cohort included a substantially higher proportion of non-survivors than the multicentre population evaluated by Cridge et al. Interestingly, CAPCSI scores did not correlate with the hematological ratios. These findings suggest that, although some hematological markers and CAPCSI were associated with mortality, they may reflect different facets of the disease.

Nevertheless, the objective of the present study was not to compare laboratory biomarkers with clinical severity scoring systems, but to determine whether HRR and RAR improved upon the prognostic information provided by their individual components. These biomarkers are not intended to support the diagnosis of AP, which should continue to rely on the integration of clinical, laboratory, and imaging findings. Rather, their potential value lies in prognostic assessment once the diagnosis has been established.

From a clinical standpoint, the cut-offs identified for RDW and albumin in the AUROC analysis fell within their respective reference intervals, and the hemoglobin threshold was only marginally below the reference range; therefore, these values would not typically stand out on a routine haemogram, but our findings suggest that they may contribute to the identification of patients at increased risk. However, these potential prognostic thresholds require external validation before clinical application. Additionally, because these measurements may vary according to analytical platform and methodology and given their only moderate discriminatory performance, all cut-offs reported here should be interpreted within the context of the equipment and population from which they were derived and considered alongside the overall clinical assessment.

Our study has several limitations. First, its retrospective, single-center design means that these results should be regarded as hypothesis-generating rather than confirmatory, particularly given the number of non-survivors and the width of the confidence intervals obtained. Prospective multicentre studies will be required to validate these findings. Although multivariable analysis was performed, the sample size may have limited its ability to detect weaker independent effects of individual biomarkers. Second, the diagnosis of AP was based on clinical signs, pancreatic lipase activity and ultrasonographic findings rather than pancreatic histopathology, the current gold standard (2). Although the use of the laboratory's standard upper reference limit (>160 U/L) rather than a higher diagnostic threshold may have permitted the inclusion of some non-specific lipase elevations, this risk was reduced by requiring concurrent clinical and ultrasonographic evidence of AP. Third, the control group comprised only 10 dogs and was not age-matched to the AP cohort. While this allowed descriptive comparisons, it was insufficient for the establishment of reference intervals and may have introduced bias, particularly because RDW can vary with age (11). Fourth, overt gastrointestinal hemorrhage was present in only 5.0% of dogs, limiting our ability to definitively assess the contribution of visible blood loss to the hematological alterations observed. In addition, all cut-offs were derived from a single analytical platform, which may limit their generalisability to laboratories using different equipment or methodologies. Finally, treatment was not standardized, and variations in clinical management may have influenced outcome independently of the biomarkers evaluated. Similarly, although euthanasia was restricted to patients considered to have terminal disease, clinical judgement inevitably contributed to these decisions. The referral nature of our hospital and the severity of cases presented may also limit the applicability of these findings to primary-care populations. Future studies should also compare these biomarkers with dogs affected by other inflammatory disorders and by anemia of different origins in order to determine whether the observed associations are specific to acute pancreatitis.

## 5 Conclusion

To the best of our knowledge, this is the first study to evaluate the prognostic utility of the RDW-based ratios HRR and RAR in dogs with AP. Although these composite índices show promise in human critical care, the combination of RDW with hemoglobin or albumin did not improve prognostic performance beyond that provided by the individual variables. Although RDW, hemoglobin, albumin and HRR were significantly associated with outcome, hemoglobin showed the highest discriminatory performance among the laboratory markers evaluated, while RAR showed no prognostic utility. Furthermore, increasing RDW values and decreasing hemoglobin, albumin, and HRR values were associated with higher mortality. Likewise, values exceeding the identified cut-off for RDW and values below the respective cut-offs for hemoglobin, albumin, and HRR were associated with an increased risk of death. However, all hematological biomarkers demonstrated only moderate prognostic accuracy. Among the variables examined, CAPCSI was the only factor independently associated with survival in the multivariable analysis, emphasizing the importance of overall clinical severity assessment. Therefore, erythrocyte-derived biomarkers may provide useful adjunctive prognostic information in dogs with acute pancreatitis, but they should be interpreted in conjunction with clinical findings and severity scoring systems rather than used as standalone prognostic tools.

## Data Availability

The datasets analyzed in this study are not publicly available due to institutional privacy policies and client confidentiality. De-identified data can be provided by the corresponding author upon reasonable request, pending authorization from the institutional data owner.
